# Lower plasma p‐tau levels and larger hippocampi may protect African American SuperAgers from age‐related cognitive decline and Alzheimer’s disease

**DOI:** 10.1002/alz.091490

**Published:** 2025-01-09

**Authors:** Wiktoria Piaszczynska, Miray Budak, Bernadette A. Fausto, Payton G. White, Robert Perna, Patricia Fitzgerald‐Bocarsly, Fanyu Hercules Tawe, Nicholas J. Ashton, Kaj Blennow, Henrik Zetterberg, Mark A. Gluck

**Affiliations:** ^1^ Rutgers University–Newark, Newark, NJ USA; ^2^ Rutgers New Jersey Medical School, Newark, NJ USA; ^3^ Department of Psychiatry and Neurochemistry, Institute of Neuroscience and Physiology, The Sahlgrenska Academy, University of Gothenburg, Mölndal, Gothenburg Sweden; ^4^ Department of Psychiatry and Neurochemistry, Institute of Neuroscience and Physiology, The Sahlgrenska Academy, University of Gothenburg, Mölndal Sweden

## Abstract

**Background:**

Advancing age is typically associated with a decline in memory capacity and increased risk of Alzheimer’s disease (AD)^1^. However, there is a subgroup of the older adult population defined as *SuperAgers* that presents excellent memory performance reflecting resilience to the typical and pathological pathways of aging ^2^. Atrophy in hippocampal subregions, key areas for memory formation, are early anatomical markers of preclinical AD^3^. Plasma p‐tau levels, particularly p‐tau 231, also show sensitivity to preclinical AD neuropathology.^4^ This study aimed to investigate structural neuroimaging and plasma biomarker differences between the SuperAger phenotype and typical agers among African Americans.

**Method:**

Participants were drawn from the ongoing longitudinal study, *Pathways to Healthy Aging in African Americans* conducted at Rutgers University–Newark. 100 participants (matched for SuperAger and typical ager status) completed a neuroimaging visit and a blood draw for plasma biomarkers. AFNI and ANTs were used for the segmentation of the medial temporal lobe and hippocampal regions of interest. Furthermore, plasma aliquots were sent for p‐tau 231 processing via Quanterix Simoa™.

**Result:**

SuperAgers had lower levels of p‐tau 231, (*t*(19) = 2.85, *p* = 0.010, *d* = 20.38) than typical agers suggesting reduced AD neuropathology burden. African American SuperAgers also had greater DG/CA3 volumes, (*t(19) = ‐2.756*, *p* = 0.013, *d* = 46.42) than typical agers.

**Conclusion:**

SuperAgers demonstrated larger hippocampal subregions implicated in memory formation and lower plasma p‐tau levels suggesting potential neuroprotective mechanisms in this unique population. SuperAgers may represent a valuable model for understanding cognitive resilience and for developing novel therapeutic strategies to mitigate age‐related cognitive decline and AD.

